# Experimental approaches to assess the effect of composition of abrasives in the cause of dental microwear

**DOI:** 10.1098/rsos.211549

**Published:** 2022-06-08

**Authors:** Matthew C. Mihlbachler, Frances Rusnack, Brian Lee Beatty

**Affiliations:** ^1^ Department of Anatomy, New York Institute of Technology College of Osteopathic Medicine, Old Westbury, NY, USA; ^2^ Division of Paleontology, American Museum of Natural History, New York, NY, USA; ^3^ United States National Museum, Smithsonian Institution, Washington, DC, USA

**Keywords:** dental microwear, experimental animals, surface metrology, abrasives

## Abstract

Dental microwear is used to investigate feeding ecology. Animals ingest geological material in addition to food. The full effect of geological abrasives on tooth wear is unknown. To evaluate mineralogical abrasives as tooth wear agents, rats were fed food manufactured with quartz silt, diatomaceous earth, and calcium carbonate. Rats were assigned to treatments and fed for 15 days. Molars were scanned with a Sensofar Plu Neox confocal microscope and evaluated using ISO-25178-2 parameters and traditional microwear variables using light microscopy. Using a pellet-diet as the control, all treatments had influence on microwear and discriminant function analyses indicated that unique surface textures had been produced. ISO variables with high discriminatory values were correlated to scratch and pit frequencies, but more ISO parameters identified changes associated with numbers of scratches than changes associated with pits. The microwear changes associated with the abrasive inclusions were co-dependent on the type of diet that the abrasives had been added to. The abrasives had less effect with pellets but produced more modified and more differentiated microwear when added to the transgenic dough. Although abrasives produce distinctive surface textures, some knowledge of the properties of food with the abrasives is needed to identify the abrasive agent.

## 1. Introduction

The primary functions of teeth are to acquire food, break down food and withstand the wear associated with these activities [1]. While developmental tooth morphology is evidence of deep adaptation for diet, changes to tooth morphology that occur during the life of the animal as a consequence of wear, as defined by the loss of tooth material following plastic deformation from indentation [2], constitute a trace record of the interactions of individual organisms with their proximate environments [3,4]. In nature, both gross wear (e.g. mesowear) and microwear are strongly associated with diet [5–8]. Nonetheless, tooth wear is a complex and incompletely understood process involving mechanical interaction of teeth and objects sampled from the environment. While analysis of dental wear has widely been used to interpret aspects of feeding ecology, the relative influences of the properties of the animal (behaviour, biomaterials) and the properties of ingesta on tooth wear have only begun to be unraveled [4,8–14].

Food is not the only ingested wear agent. In herbivores, opal phytoliths are most often the most abrasive food particles, which are silica bodies formed by plants, possibly to protect their vascular bundles from herbivores. Depending on myriad environmental variables (e.g. aridity, soil composition, stocking rate, feeding height) animals ingest sediment, commonly referred to as ‘grit’ [9,10,15–17]. In order to robustly test hypotheses of dietary ecology with dental wear, a better understanding of how geological materials of different mineralogies contribute to dental wear and what wear patterns they leave is needed. High rates of ingestion of sediments can lead to excessive and pathological dental wear [18–20] and intervals of high grit consumption during certain periods of earth history may have driven the evolution of dentitions and adaptations for wear resistance in numerous lineages of rodents, ungulates, and other extinct clades [15]. Among rodents, in particular, palaeodietary analyses may be especially valuable as palaeoenvironmental indicators because rodents typically have small home ranges, including *Rattus norvegicus* [21–24]. A greater understanding of how environmental and dietary conditions relate to microwear might allow the use of fossil rodent dentitions to determine local environmental conditions with greater accuracy than the teeth of animals with larger home ranges that may reflect wear from a variety of locations.

*In vitro* attempts to abrade enamel with both biogenic and geological abrasives have demonstrated that both kinds of materials can wear teeth and produce microwear features [2,12,25–34]. In investigations of dental wear rates, Müller *et al*. [35] fed domestic rabbits and guinea pigs pellet diets with organic abrasives (grass and rice hulls with phytoliths) and quartz sand. Organic abrasive agents (grass and rice hulls containing phytoliths) had measurable effects on dental wear rates, but the inclusion of sand greatly accelerated the wear rate of cheek teeth. In treatments with only organic abrasives, rabbit cheektooth (P3) wear was approximately 1 mm per week, while with sand, P3 wear was 4–5 mm per week [35].

It is clear that both organic and inorganic components of ingesta cause wear. However, a consensus on which of these wear agents is more commonly the primary contributor to dental wear in nature and to what degree either have been an agent for evolution of dental morphologies (e.g. hypsodonty) has not emerged [36]. Moreover, a consensus on if and how food and grit differentially affect microwear has not yet emerged [4,12,14,37–39]. High-abrasion microwear patterns, such as excessive scratches or pits, could be caused by hard and tough food items, ingestion of phytoliths, a high rate of grit ingestion due to feeding near the ground, or some combination of these factors. Sand and silt grade particles of silicate minerals, primarily quartz and feldspar are the primary geological abrasive agents that may contaminate diets of wild terrestrial vertebrates [40]. However, other minerals occur in some regions, including calcium carbonate and/or diatomaceous earth substrates of limestone-dominated bedrock in passive continental margins such as peninsular Florida, parts of Central America and the coast of the United Kingdom [41–44].

Some studies have introduced mineralogical abrasives into animal diets to measure their effects on microwear. Baines *et al*. [45] introduced a quartz sand substrate to fish with non-occluding teeth and found the abundance and proximity of discrete wear scars (microwear features) to be the most dramatically affected microwear variable. Covert and Kay [46] fed opossums a soft cat food diet with treatments containing plant fibres, chitin and fine-ground pumice. Although the duration of this study was of sufficient duration to produce dental wear [47], only the pumice-fed animal had altered microwear with many parallel striations, resembling grass eating hyraxes [48]. The results of this study were questioned [49] because the soft cat food matrix did not mimic natural diets. Teaford & Lytle [50] found that different grit mineralogies led to differences in human dental wear rate with sandstone-ground maize producing a higher rate of microwear feature accumulation compared with maize ground with igneous rock. Hoffman *et al*. [10] fed goats fine- and medium-grained quartz sand with Garrison and Brome hay (which also contains biogenic silica). There was a significant increase in pit features correlated with an increase in grain size. Merceron *et al*. [39] fed sheep clover- and grass-dominated hay with added silica and feldspar dust. Adams *et al.* [17] found no significant differences between microwear signatures of moles and bats, despite the quartz sand outside and inside the bodies of mole diets and the relatively clean bat diets. Significant differences were found between the food types but not between dusty and dust-free diets. These experimental results involving sheep do not agree with observations made in natural feeding scenarios—the microwear of naturally grazing sheep has in some scenarios been described as pitted, while the teeth of sheep that have a high soil consumption rate are more highly striated [51]. Subsequent experimental studies on sheep fed different diets with different concentrations of abrasives have demonstrated the inclusion of sand exerts influence on microwear [4,14]. *In vitro* studies of exogenous abrasives added to foods with different textures has shown that the material properties of foods affect how the abrasives added to them can cause wear [52]. An additional concern is that food texture and abrasives may influence masticatory behaviours in ways that would naturally change the varying degrees of orthal versus translational movements employed [53], which may result in differing wear textures.

**Figure 1.**
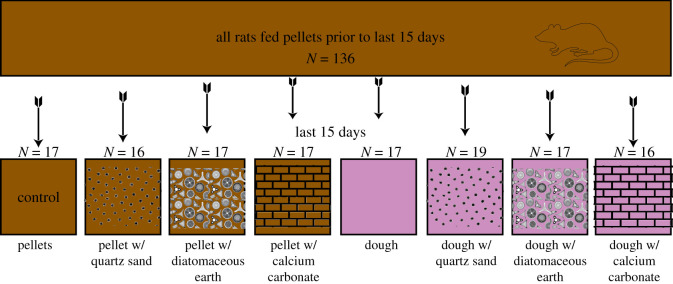
Outline of basic research design where rats were fed seven experimental diets and compared with a control diet composed of standard pellet chow.

The above studies used a wide variety of methods including traditional microwear methods (TM) where a human observer counts and classifies discrete microwear features according to size and shape (pits and scratches), and more recently developed dental microwear texture analysis methods (DMTA) involving confocal and focus variation microscopy. While most recent DMTA studies use variables that until recently were proprietary [54], standardized parameters ISO 25178-2 [55] have become available and are increasingly used to study dental microwear [4,9,14,36,56–60]. The development of ISO 25178-2 introduces a set of standardized parameters with which dental microwear textures can be investigated. This paper reports on experiments where geological abrasive particles (quartz silt, diatomaceous earth and calcium carbonate), were added to the diets of rats (figure 1). The resulting microwear was analysed with ISO 25178-2 surface texture parameters (ISO-based DMTA and TM. There have been relatively few studies comparing ISO-based DMTA with TM. TM studies attempt to recognize discrete microindentations and categorize them according to shape (e.g. pits and scratches) but ignores the remaining wear surface (between the visible indentations). ISO 25178-2 characterizes the texture of an entire wear surface but does not recognize discrete microindentations. Merging the two may be the best way to compensate for the respective imitations of these two methods.

We attempt to relate ISO parameters and TM variables to wear patterns caused by these mineralogical abrasives and identify wear signals that may be indicative of wear due to ingestion of geological materials. To control for abrasion from the food itself, some rats were fed a standard pelleted rat chow diet, while others were fed a soft transgenic dough. Abrasive particles were added to both diets (figure 1). Quartz is significantly harder (5.4–13.7 GPa) [61] than observed values of the hardness of mammalian enamel (0.2–0.35 GPa) [36,62]. Naturally occurring calcium carbonate (CaCO_3_) from invertebrate sources is often softer than enamel, but can have a range of hardness values 1.5–19.32 GPa, depending on the mineralogy and structure [63]. Diatomaceous earth has a variety of reported hardness values that are likely to be due to the complex structure of diatoms themselves (0.043–1.7 GPa) [64]. Quartz is predicted to have the highest influence on microwear because the mineral hardness of SiO_2_ is consistently greater than that of hydroxyapatite, the material from which enamel is composed. Thus, if isolated within a soft diet like the transgenic dough diet, the quartz silt group would hypothetically demonstrate the greatest number of differences in ISO parameters from other groups. Considering how the material properties of the foods' materials have an influence on the impact the abrasives can have [52], this effect should be greater in the dough diet largely because of the lack of the dampening effect of similar material properties in the pellet diet. Calcium carbonate and diatomaceous earth have hardness values generally less than enamel and are not expected to contribute microwear evidence of abrasion to the same degree as quartz. Thus, we hypothesize that the ISO parameters will not be significantly different between pellet diet and those with added diatomaceous earth or calcium carbonate. Although material hardness is not the only property of abrasive particles that determine the generation of wear [2], it has a significant impact on the extent and type of wear that will occur [12,30,33,65,66].

## 2. Material and methods

### 2.1. Experimental animals and specimen preparation

This study sample consists of 138 specimens of the Long Evans strain of *Rattus norvegicus.* Controlled feeding experiments were conducted at NYIT-College of Osteopathic Medicine with approval from the university Institutional Animal Care and Use Committee (IACUC protocol 2014 BB 01). All rats were male and were 35 days old when received from the animal breeder (Charles River), ensuring that the upper second molar was in eruption for the duration of the experiment [67].

The fact that animals naturally accumulate pre-existing microwear presented an unavoidable problem. Prior to the experiment, rats had been fed their normal pelleted rat chow diets for their entire lives after being weaned. Our control treatment in which rats were continued on the same pellet diet (without mineralogical inclusions) verified that the standard pellet diet generated pre-existing dental microwear. The rats were divided into eight varying diet categories. Rats were first designated to either a chow (pellet form) diet (Rodent Diet, grain-based) or bacon dough (soft form) diet (Transgenic Dough Diet bacon flavour) (both are custom-modified foods produced by Bio-Serv, Flemington New Jersey). Within each food type, rat diets were further subdivided into four groups: (1) no added abrasives, (2) calcium carbonate (grain size 10–50 µm, produced by L. D. Carlson, Kent, Ohio), (3) diatomaceous earth (grain size 10–40 µm, food grade diatomaceous earth produced by DiatomaceousEarth.com) and (4) quartz silt (grain size 45–55 µm, commercially available pool filter ‘sand’, produced by HTH) (figure 1). Pellet form food contained 7% dry ash, and the transgenic dough contained 3.3% dry ash prior to adding abrasives. For each modified food, approximately 5% of food volume was added abrasives.

Rats were exposed to these special diets for 15 days and were then sacrificed. All diets were provided to the rats at the same volume daily, though it superficially appeared that transgenic dough diets were consumed faster than pelleted diets. Rats were euthanized using carbon dioxide and guillotine. Heads were removed for ease of dissection (further detail available through IACUC protocol 2014 BB 01). The rat heads were bisected and the right side of each was used for this study. The right maxilla and mandible of each specimen were separated. The teeth were not removed from the maxillary and mandibular bones. The bones and teeth were placed in open containers and allowed to desiccate in refrigerated air for two to three weeks.

After desiccation, adhering muscle, periosteum and fascia were removed with forceps with care not to contact teeth with forceps. All teeth were first cleaned with 91% isopropyl alcohol by cotton swab. Up to four additional cleanings were conducted using a dish soap and water mixture (1 : 20 mixture by volume). Cotton swabs were used to apply the mixture to the teeth in a circular motion for approximately 20 s. Teeth were then rinsed under warm running water for approximately 20 s. The teeth were laid to dry to completion. If imaging demonstrated remaining debris, the cleaning process was repeated until satisfactory.

### 2.2. Surface metrology

**Figure 2.**
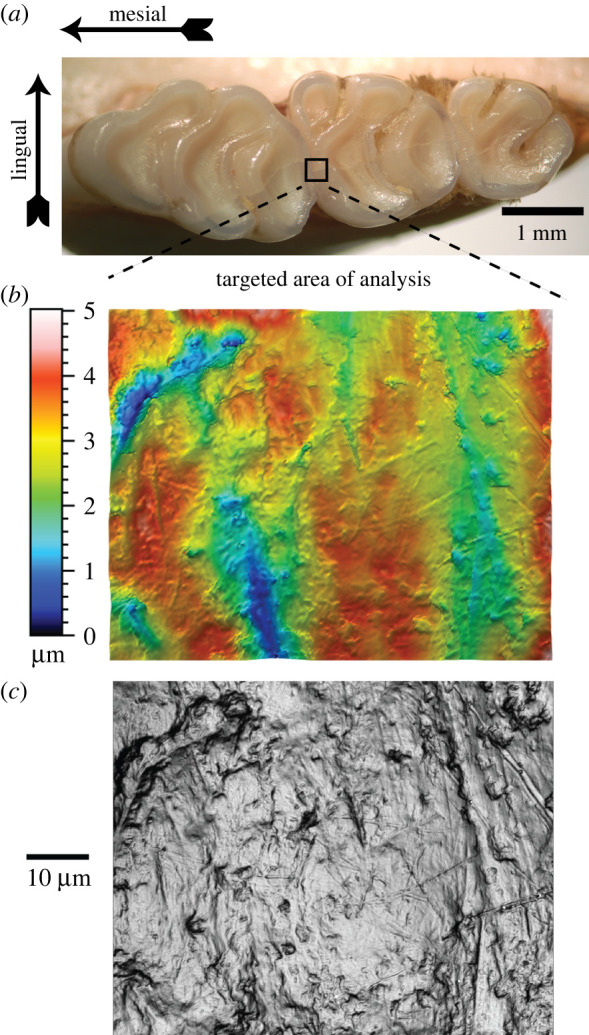
(*a*) Upper molar row showing targeted area of analysis on the right upper second molar; (*b*) colour topography map with colour Z scale; (*c*) greyscale photo simulation of the same surface.

The Sensofar Plu NEOX optical profiler was used to image the teeth using SensoScan software. In an earlier paper Mihlachler *et al.* [68] we used these same teeth to compare data from teeth with casts made from polyvinylsiloxane moulds. That paper focuses on differences on the effect of data loss in replication. This paper uses only data from the teeth themselves, and reports on the relationship of wear with the various abrasives. Images were obtained of the mesial enamel ridge of each specimen's second maxillary molar (M2) (figure 2*a*). The maxilla was positioned using clay and placed on the microscopy field with M1 positioned anteriorly. Z-level was adjusted to bring the specimen into focus. An initial 4 × 5 overview image (4 rows × 5 columns of orientation images = a montage of 20 images) was obtained to grossly orient the specimen. Using the overview image as guide the middle area of the M2 mesial enamel ridge was targeted. Within the SensoScan program, the corresponding objective was selected (150×, numerical aperture = 0.9). ‘Autolight’ was used to adjust the exposure. With the ‘+’ over the intended enamel ridge, the Z-level was increased to bring the image completely out of focus and the reference was defined. The Z-level was then decreased to bring the image completely out of focus in the opposite direction. The relative position of Z was used to determine the range for the Z-scan and the single Z-scan definition was set as ‘bottom’ up. Each Z step size = 0.1 µm, and the spatial sampling at 150× = 0.09 µm pixel^−1^. The scan was then acquired as a .plu file using confocal settings with white light, which was then imported into SensoMAP as a ‘studiable’. Scans were rejected and redone if recovery of surface was less than 90%.

Each studiable was loaded into SensoMAP Premium software (v. 7.2.7368) in order to process the image and attain surface texture measurements. An area of 80 × 100 μm was extracted using the ‘extract area’ operator. Extracted data was then levelled using the ‘least squares planes' method and form was removed using ‘polynomial of degree 3’. Data were processed through a Gaussian filter. Pseudo-colour view of the surface image (figure 2*b*) was converted to three-dimensional view. Amplification of the three-dimensional image was normalized in X, Y and Z. The normalized three-dimensional image was then manipulated and viewed in various dimensions to visualize areas of excessive, abnormal peaks residue. These problem areas were retouched using the ‘retouch surface points' operator in a 2 × 2 μm ellipse and filled with non-measured points. Missing data points were filled in using the ‘fill in non-measured points’ operator set to replace points by ‘a smooth shape calculated from the neighbours’. The three-dimensional image was generated and viewed again to ensure resolution of problem areas. If required, the problem areas were retouched again through the same process. Parameters table and texture direction studies (table 1) were then obtained and numerical results were exported as a CSV file. The SensoMAP worksheet was saved and a PDF copy was created. The process was continued for each specimen's 150× images.

**Table 1.** ISO surface metrology parameters and results of ANOVAs comparing means of the eight diet treatments. Italics indicate significant differences for parametric analysis of raw data values (Sig. P) and non-parametric analysis of rank-transformed data (RT Sig. P).

parameter name	Explanation	units	F	Sig. P	RT Sig. P
height parameters					
Sq	root-mean-square-height	μm	5.868	*0.000*	*0.000*
Ssk	skewness		1.397	0.212	*0.036*
Sku	kurtosis		1.105	0.364	*0.446*
Sp	maximum peak height	μm	6.673	*0.000*	*0.000*
Sv	maximum pit height	μm	4.712	*0.000*	*0.000*
Sz	maximum height	μm	5.872	*0.000*	*0.000*
Sa	arithmetic mean height	μm	6.053	*0.000*	*0.000*
functional parameters					
Smr	areal material ratio	%	4.94	*0.000*	*0.000*
Smc	inverse areal material ratio	μm	6.383	*0.000*	*0.000*
Sxp	extreme peak height	μm	4.275	*0.000*	*0.001*
spatial parameters					
Sal	autocorrelation length	μm	0.673	0.694	0.816
Str	texture-aspect ratio		1.27	0.270	0.436
Std	texture direction	°	0.758	0.624	0.165
hybrid parameters					
Sdq	root-mean-square gradient		11.614	*0.000*	*0.000*
Sdr	developed interfacial area ratio	%	12.158	*0.000*	*0.000*
functional parameters (volume)					
Vm	material volume	μm^3^ μm^−^^2^	3.678	*0.001*	*0.003*
Vv	void volume	μm^3^ μm^−2^	6.432	*0.000*	*0.000*
Vmp	peak material volume	μm^3^ μm^−2^	3.805	*0.001*	*0.002*
Vmc	core material volume	μm^3^ μm^−^^2^	5.916	*0.000*	*0.000*
Vvc	core void volume	μm^3^ μm^−2^	6.484	*0.000*	*0.000*
Vvv	pit void volume	μm^3^ μm^−^^2^	3.385	*0.002*	*0.004*
feature parameters					
Spd	density of peaks	1 μm^−^^2^	2.013	0.058	0.056
Spc	arithmetic mean peak curvature	1 μm^−1^	8.071	*0.000*	*0.000*
S10z	ten point height	μm	5.285	*0.000*	*0.000*
S5p	five point peak height	μm	3.253	*0.003*	*0.001*
S5v	five point pit height	μm	4.429	*0.000*	*0.000*
Sda	mean dale area	μm^2^	0.962	0.462	0.169
Sha	mean hill area	μm^2^	2.055	0.053	*0.049*
Sdv	mean dale volume	μm^3^	0.968	0.458	0.088
Shv	mean hill volume	μm^3^	1.16	0.330	0.666
other					
isotropy			1.27	0.270	0.436
first direction			1.287	0.262	0.070
second direction			0.588	0.765	0.667
third direction			1.089	0.374	0.241

### 2.3. TM methods

Photosimulations that resemble greyscale images of TM microwear (lighting height at 45° and azimuth angle of 90°) (figure 2*c*) derived from the methods described (after all data processing, including levelling, filtering and form removal) above were analysed with a TM method described by Mihlbachler *et al*. [69] and Mihlbachler & Beatty [70]. The original photosimulations (1338 × 1070 pixels) were resampled in Adobe Photoshop to reduce the number of pixels down to 20% of the original pixel density (268 pixels × 214 pixels: 7.18 pixels µm^−2^ of tooth surface area).

Microwear features were assigned to one of four primary categories as defined in table 3. Two secondary variables were calculated: TS (total number of scratches) and TP (total number of pits). Microwear features smaller than the above criteria were not counted because observer repeatability for small, poorly resolved microwear features was found to be low with similar methods [70].

The images from which TM data were collected were randomly ordered and named with arbitrary numbers. Therefore, the observer (M.M.) was blind to the diet treatment to which each specimen belonged. Microwear features were identified and traced directly on the images by superimposing standardized circles (for pits) and lines (for scratches) in Adobe Illustrator. The images were examined in random order three times by a single observer. The superimposed tracings were saved after each pass, preserving the observer's interpretation. Multiple passes allows the images to be more comprehensively sampled for discrete microwear features by eliminating the diminishing effects of observer fatigue. During each pass, additional features that had been missed in earlier passes were identified. By the third pass, very few additional microwear features were recognized (one or two per image) so additional passes were not made.

### 2.4. Statistical methods

The study produced data on 34 ISO parameters (table 1 and electronic supplementary material) and six TM variables (four primary variables and two secondary variables) (table 3) that were analysed separately and together. All statistical analyses were performed on SPSS v. 24. For brevity, ISO parameters and TM variables are collectively referred to as ‘variables’ in many instances below. To test for differences in the mean values for each of these variables among the eight dietary groups, one-way ANOVAs were performed. Most of the data have distributions that are not significantly different than normal, although in some feeding treatments some variables tested significantly for a non-normal distribution (Shapiro–Wilk *p* ≤ 0.05). ANOVA is empirically shown to be robust to violations of the assumption of normal distribution [71,72]. However, to ensure that violations of normality had no major influence on our results, we ran additional ANOVAs on the rank-transformed ISO data. The differences between these analyses are detailed below. Levene's test was used to test for unequal variances. For most variables, equal variances could not be falsified. Tukey's *post hoc* tests were used to determine which of the numerous two-way comparisons of the analyses of raw data significantly differ. For variables found to have unequal variances (Sv, Smr, Sdr) we used Dunnett's T3. In the results section, we focus mainly on two-way comparisons between the control diet (pellets) with the other diet treatments. The expanded results, in which every possible two-way comparison is tested between every variable and every dietary treatment, are provided in the electronic supplementary material.

To evaluate potential relationships between the TM and ISO variables, Pearson correlation coefficients were calculated. In the results section, we focus on comparisons of ISO and TM data; however, the expanded results (with correlation coefficients calculated between all variables) are provided in the electronic supplementary material. Keeping in mind the large number of statistical tests reported below, we caution that the distributions of significant results within the ISO and TM datasets are more meaningful than the results of any of the individual tests.

To measure the success of ISO and TM variables at predicting membership within each diet treatment, three discriminant function analyses were performed: (1) ISO data using all parameters, (2) TM data, using primary variables (WS, NS, SP, SL), and (3) and a ‘total evidence’ analysis that combined ISO (all variables) and TM data (primary variables). Discriminant function analyses (DFAs) of raw data and rank-transformed data are reported in table 6. Wilks' lambda indicates the power of the discriminant function (DF) to distinguish between the diet treatments with lower values indicating a more powerful DFA. The chi-square statistic corresponds to Wilks’ lambda and, if statistically significant, indicates a significant relationship between the dependent groups and the independent variables. We also report the percentage of correctly classified specimens. Although there is not a direct relationship between Wilks' lambda and the accuracy of the classifications based on the discriminant functions, both are potentially good measures of the power of the DFA.

Finally, calculations were made to compare the total discriminating power of each variable. The following calculation summarizes the total influence each variable, considering the diminishing importance of each subsequent DF, where *c* equals absolute values of canonical loadings (table 7), *v* equals the proportion of variance explained by each DF (table 7) and *N* is the number of discriminant functions (*N* = 7).

$$\overset{N}{\underset{i=1}{\sum }}c^{i}v^{i}.$$

## 3. Results

### 3.1. ANOVAs

**Figure 3.**
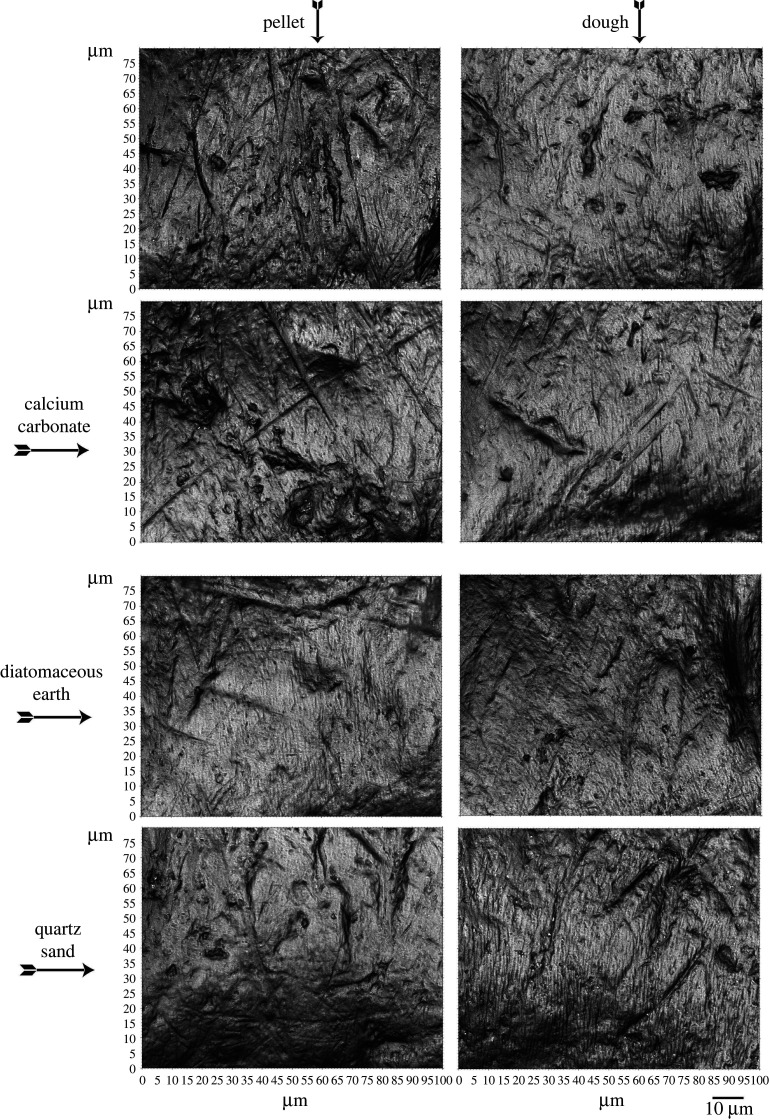
Greyscale photo simulations for each feeding treatment. The shown images display numbers of pits and scratches that are near the mean for each treatment (figure 4) are roughly representative of the average surfaces of each feeding treatment.

**Figure 4.**
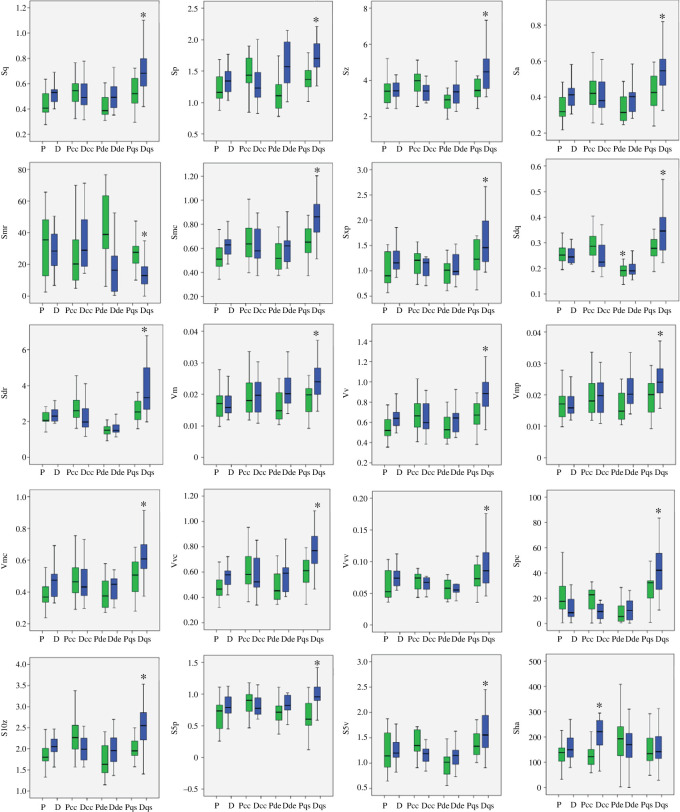
Box and whisker plots of individual ISO parameters for each feeding treatment. Asterisk denotes significant differences from other feeding treatments according to Tukey's *post hoc* tests (table 2). Only those parameters with significant results are included. Green boxes are pellet diets, blue boxes are dough diets.

The 15-day exposure of rats to eight dietary treatments produced a variety of visibly different surface textures (figure 3) for which significant differences were found in the ISO and TM data. 20 out of 34 ISO variables showed statistically significant differences based on ANOVAs using raw data (table 1 and figure 4). Significant differences were found among most families of ISO parameters, including height parameters, functional parameters, hybrid parameters and feature parameters. No significant differences were found among spatial parameters and other parameters. Analysis of rank-transformed data provided comparable results, although Ssk produced significant results in the analysis of rank-transformed data (*p* = 0.036) but not in the raw data (*p* = 0.212). For Sha, the significance differences between the two tests were trivial (*p* = 0.053 for raw data and *p* = 0.049 for rank-transformed data). In this case, rejection or acceptance of the null hypothesis for Sha would seem arbitrary, although a significant pairwise difference between two treatments (*p* and Dcc) for Sha was found in the *post hoc* results (table 2). Significant differences between the feeding treatments were also found among the TM variables except WS (wide scratches) (table 3).

**Table 2.** Tukey's test *p*-values for pairwise comparisons of ISO parameters for which significant differences were found between the pellet diet control group and other diet groups. Italics indicate significant results. D = dough; Dcc = Dough w/ calcium carbonate; Dde = Dough w/ diatomaceous earth; Dqs = Dough w/ quartz silt; P = pellet; Pcc – Pellet w/ calcium carbonate; Pde = Pellet with diatomaceous earth; Pqs = Pellet w/ quartz silt. Table 1 for ISO parameter abbreviations. Parameters with no significant results are not shown.

pellet versus	Sq	Sp	Sz	Sa	Smr	Smc	Sxp	Sdq	Sdr	Vm	Vv	Vmp	Vmc	Vvc	Vvv	Spc	S10z	S5p	S5v	Sha
D	0.66	1.00	1.00	0.60	1.00	0.65	0.69	1.00	1.00	1.00	0.67	1.00	0.52	0.69	0.83	0.66	0.95	0.69	1.00	0.99
Pcc	0.32	0.85	0.93	0.26	0.95	0.20	0.78	0.90	0.87	0.82	0.20	0.82	0.27	0.16	0.95	1.00	0.25	0.58	0.61	1.00
Pde	1.00	0.99	0.48	1.00	0.69	1.00	1.00	*0.04*	0.22	1.00	1.00	1.00	1.00	0.99	1.00	0.40	0.99	1.00	0.87	0.68
Pqs	0.48	1.00	1.00	0.35	1.00	0.41	0.54	0.96	0.96	1.00	0.42	0.98	0.28	0.43	0.79	0.94	0.98	1.00	0.84	1.00
Dcc	0.66	1.00	1.00	0.54	1.00	0.51	0.93	0.99	1.00	0.93	0.51	0.93	0.44	0.46	0.98	0.76	0.99	0.62	1.00	*0.05*
Dde	0.87	0.14	1.00	0.83	0.22	0.73	1.00	0.07	0.25	0.41	0.70	0.42	0.85	0.61	1.00	0.86	1.00	0.54	1.00	0.88
Dqs	*0.00*	*0.00*	*0.00*	*0.00*	*0.03*	*0.00*	*0.00*	*0.00*	*0.00*	*0.00*	*0.00*	*0.00*	*0.00*	*0.00*	*0.00*	*0.01*	*0.00*	*0.01*	*0.03*	1.00

**Table 3.** Traditional microwear (TM) variables and results of ANOVAs comparing means of the eight diet treatments. Italics indicate significant differences.

variable	abbreviation	explanation	*F*	Sig
narrow scratches	NS	max width = 1.25–2.5 μm	9.336	*0.000*
wide scratches	WS	max width > 2.5 μm	1.776	0.098
small pits	SP	max diameter 2.5–5 μm	3.869	*0.000*
large pits	LP	max diameter > 2.5 μm	7.929	*0.000*
total scratches	TS	NS + WS	8.196	*0.000*
total pits	TP	SP + LP	7.238	*0.000*

ISO data found significant differences between the pellet control (P) and the treatments Pde (pellet with diatomaceous earth), Dcc (dough with calcium carbonate) and Dqs (dough with quartz silt) (table 2). The majority of these differences involving 19 ISO parameters were produced by the Dqs treatment. The significant difference produced by the Pde and Dcc treatments, each involving only a single ISO parameter are suspect and possibly attributable to type II error.

**Figure 5.**
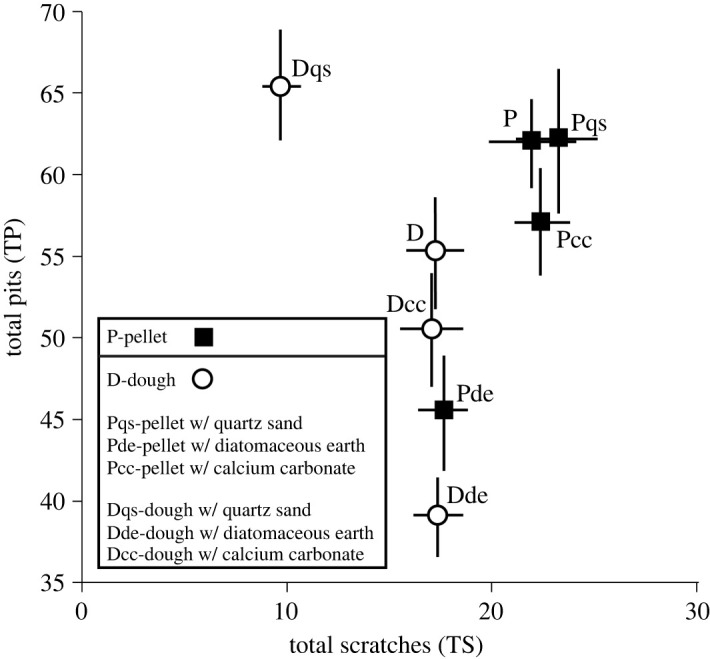
Means and standard errors of TS (total scratches) and TP (total pits).

TM found differences between the same pairings but produced additional significant differences between P and Dde (dough with diatomaceous earth) (table 4). Plots of ISO data distributions (figure 4) overwhelmingly suggest Dqs experienced the most extensively altered surface texture. Plots of TM data, however, produced results that suggest multiple diet treatments experienced major changes in dental microwear (figure 5). TM results suggest the microwear changes produced by the Dqs diet are related to a reduction in the number of observable scratches. TM data suggest Pde, Dcc and Dde experienced major reductions in the numbers of observable pits. ISO data did not identify a difference between P and the Dde treatment, although according to TM data it was the most significantly altered treatment in terms of reductions of pits. Many ISO parameters identified changes in the feeding treatment in which the number of scratches was reduced. However, ISO was less successful in finding differences in treatments in which there were significant losses to the number of pits.

**Table 4.** Tukey's test *p*-values for pairwise comparisons of TM parameters for which significant differences were found between the pellet diet control group and other diet groups. Italics indicate significant results. Table 3 for TM variable abbreviations and feeding treatment abbreviations.

pellet versus	WS	NS	SP	LP	TS	TP
D	0.97	0.11	0.98	0.77	0.36	0.85
Pcc	0.91	1.00	1	0.06	1.00	0.97
Pde	0.42	0.08	0.51	*0.00*	0.52	*0.01*
Pqs	0.27	1.00	1.00	0.96	1.00	1.00
Dcc	1.00	0.23	0.85	*0.04*	0.35	0.24
Dde	1.00	0.35	0.08	*0.00*	0.43	*0.00*
Dqs	0.99	0.00	0.91	0.91	*0.00*	1.00

### 3.2. Correlation of ISO and TM

**Figure 6.**
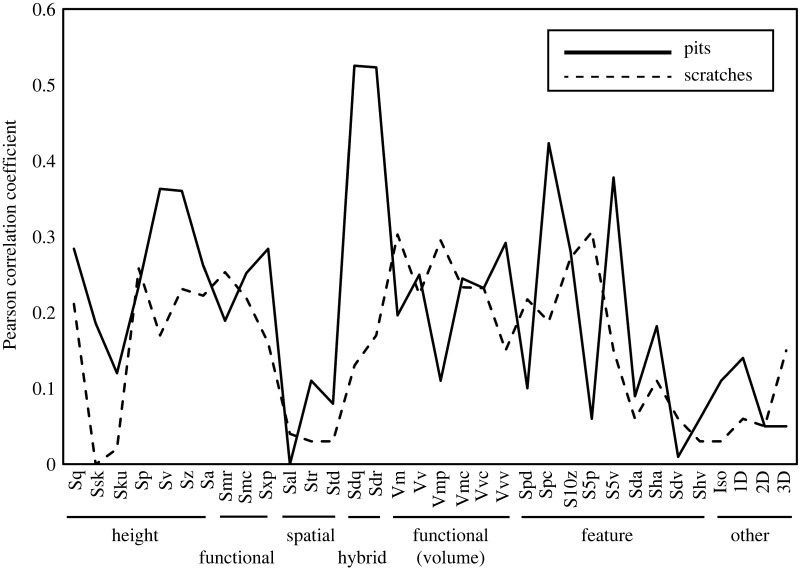
Pearson correlation coefficients comparing TS (total scratches) and TP (total pits) with ISO parameters.

Because ISO and TM data found one or more significant differences between P and a similar subset of feeding treatments (Pde, Dcc, Dqs), there ought to be a high degree of correlation between ISO and TM data, and indeed this turns out to be the case (table 5). Eighty-five out of 204 (42%) possible correlations of ISO parameters and TM variables are significantly correlated. The highest correlations are between pits and hybrid parameters (Sdq and Sdr) (figure 6). Fifty-one per cent of correlations of ISO and TM pit variables are significant (*p* ≤ 0.05) and 37% are highly significant (*p* ≤ 0.01). Thirty-three per cent of ISO comparisons with scratch variables are significant (*p* ≤ 0.05) and 24% are highly significant (*p* < 0.01).

**Table 5.** Pearson correlation coefficients comparing ISO parameters and TM variables. **indicates correlation is significant at the *p* ≤ 0.01 level (2-tailed). *indicates correlation is significant at the *p* ≤ 0.05 level (2-tailed). Table 1 for ISO parameter abbreviations.

	wide scratches	narrow scratches	large pits	small pits	total scratches	total pits
Sq	0.00	−0.235**	0.279**	0.211*	−0.211*	0.284**
Ssk	−0.02	0.01	−0.293**	−0.11	−0.02	−0.186*
Sku	−0.12	0.01	0.09	0.11	−0.02	0.12
Sp	−0.02	−0.256**	0.14	0.191*	−0.258**	0.235**
Sv	−0.07	−0.173*	0.364**	0.273**	−0.17	0.363**
Sz	−0.07	−0.231**	0.296**	0.290**	−0.231**	0.360**
Sa	0.01	−0.250**	0.277**	0.186*	−0.222**	0.262**
Smr	0.04	0.236**	−0.07	−0.16	0.253**	−0.189*
Smc	−0.02	−0.240**	0.241**	0.192*	−0.219*	0.252**
Sxp	0.04	−0.202*	0.327**	0.194*	−0.16	0.284**
Sal	−0.178*	0.00	−0.04	0.00	−0.04	0.00
Str	0.00	0.02	0.10	0.09	0.03	0.11
Std	0.14	−0.04	−0.08	−0.03	0.03	−0.08
Sdq	0.07	−0.16	0.496**	0.394**	−0.13	0.525**
Sdr	0.07	−0.197*	0.513**	0.390**	−0.17	0.523**
Vm	−0.08	−0.275**	0.07	0.17	−0.303**	0.196*
Vv	−0.02	−0.243**	0.235**	0.192*	−0.224**	0.250**
Vmp	−0.08	−0.252**	−0.01	0.10	−0.295**	0.11
Vmc	0.01	−0.259**	0.285**	0.16	−0.233**	0.245**
Vvc	−0.02	−0.251**	0.219*	0.177*	−0.232**	0.232**
Vvv	0.02	−0.180*	0.338**	0.198*	−0.15	0.292**
Spd	0.12	0.204*	0.11	0.08	0.217*	0.10
Spc	0.05	−0.216*	0.329**	0.350**	−0.188*	0.423**
S10z	−0.12	−0.248**	0.240**	0.191*	−0.272**	0.281**
S5p	−0.07	−0.300**	0.04	0.02	−0.306**	0.06
S5v	−0.10	−0.12	0.341**	0.270**	−0.15	0.378**
Sda	0.07	−0.07	−0.16	−0.05	−0.06	−0.09
Sha	−0.02	−0.11	−0.11	−0.184*	−0.11	−0.182*
Sdv	−0.04	−0.05	0.10	−0.05	−0.06	0.01
Shv	0.04	0.01	−0.01	−0.10	0.03	−0.06
isotropy	0.00	0.02	0.10	0.09	0.03	0.11
first direction	−0.08	−0.04	−0.10	−0.10	−0.06	−0.14
second direction	0.09	0.01	0.03	−0.06	0.05	−0.05
third direction	0.04	0.16	0.07	−0.08	0.15	−0.05

### 3.3. Discriminant function analysis

**Figure 7.**
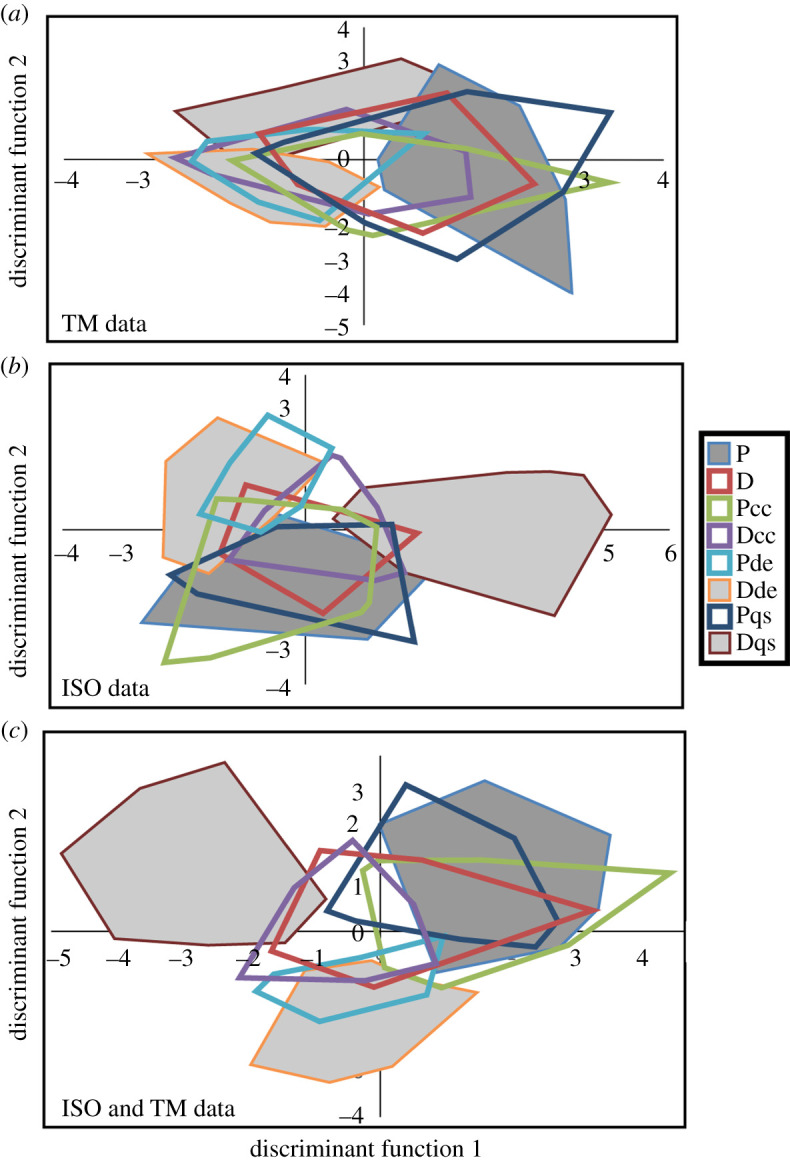
Outlines of areas occupied by the eight feeding treatments on discriminant functions one (x-axis) and two (y-axis) for three discriminant function analyses involving the TM data, ISO data, and total evidence analysis where ISO and TM data are combined. These results are based on the analysis of raw data. Expanded plots with individual data points are in the electronic supplementary material.

Discriminant function analyses of both the ISO and TM datasets were significant, as was the total evidence analysis that combined ISO and TM data (table 6). Raw data and rank-transformed data produced similar percentages of correct *a posteriori* classifications. Overall, ISO performed better than TM with higher *post hoc* classification rates and slightly better separation of the feeding treatments in figure 7*a,b*). The total evidence analysis produced the best performing DFA and successfully reclassified the highest number (71.1% for raw data, 78.5% for rank-transformed data) of specimens and most extensively separated the feeding treatments (figure 7*c*), suggesting that both ISO and TM data contained unique discriminatory information.

**Table 6.** Results of discriminant function analyses on the raw data (above) and rank-transformed data (below) involving ISO data, TM data, and ISO and TM combined data.

	chi-squared	sig. (P)	Wilks' lambda	% correctly classified	% var F1	% var F2	% var F3
raw data							
TM	136.91	*p* < 0.001	0.346	44.9	52.9	37	7.7
ISO	310.12	*p* < 0.001	0.071	65.2	38.9	20.8	16.6
ISO + TM	380.61	*p* < 0.001	0.038	71.1	43.6	22.9	12.9
RT data							
TM	140.83	*p* < 0.001	0.336	41.9	50.2	42	6.2
ISO	355.9	*p* < 0.001	0.044	68.1	34.6	22.0	15.0
ISO + TM	443.5	*p* < 0.001	0.019	78.5	34.2	25.0	15.1

**Figure 8.**
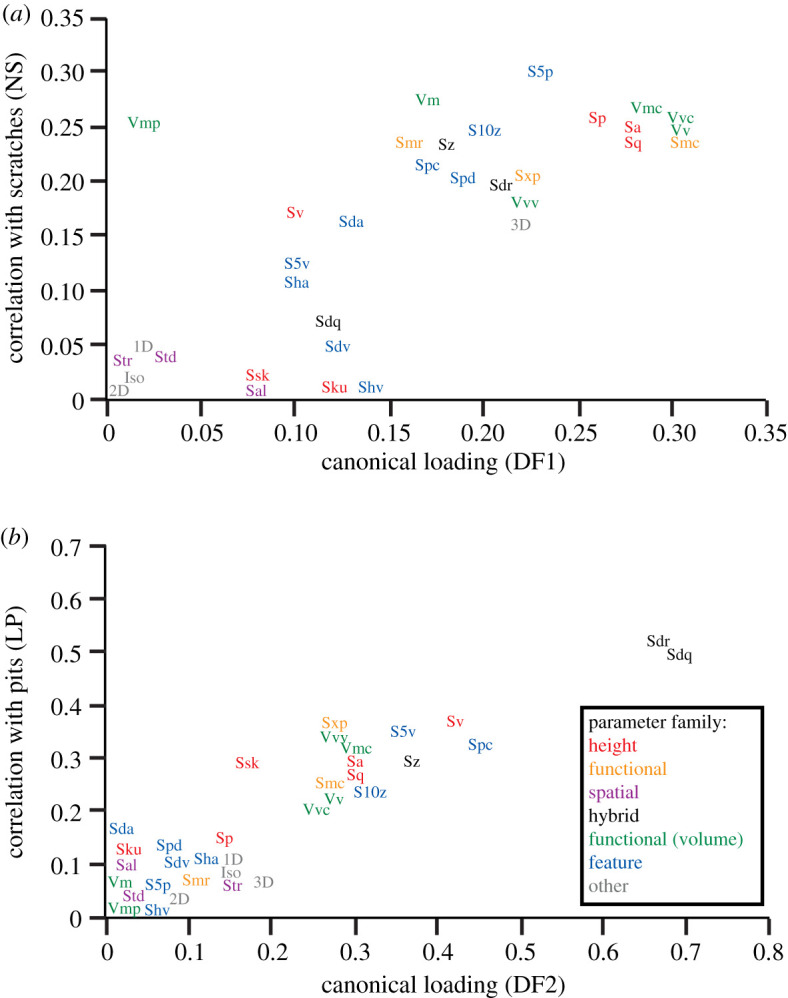
Absolute values of canonical loadings of ISO variables based on total evidence analysis of raw data versus Pearson correlation coefficients with TM variables (*a*) narrow scratches (NS) for DF1, and (*b*) large pits (LP).

The canonical structure of the data is heavily influenced by the abundances of scratches and pits. The variation in the frequencies of scratches and pits caused by the feeding treatments primarily resulted in texture differences strongly related to the classic TM microwear categories (scratches and pits) and aspects of ISO that are sensitive to these categories of microwear features. In the total evidence analysis, DF1 is most heavily influenced by scratches, as NS (narrow scratches) is the most highly loaded variable. Similarly, the loadings for ISO parameters on DF1 (table 7) are significantly related to their correlations with NS (table 5) (figure 8*a*: Pearson correlation coefficient = 0.718; *p* < 0.001). DF2 is associated with pits to an even stronger degree. LP loaded heavily on DF2 (table 5). ISO parameters loaded on DF2 (table 7) in proportion to their degree of correlation with LP in table 5 (figure 8*b*: Pearson correlation coefficient = 0.868; *p* < 0.001).

**Table 7.** Per cent of variance explained (top row) and canonical correlates for the seven discriminant functions resulting from total evidence DFA of TM and ISO raw data. Abbreviations for ISO parameters and TM variables are in tables 1 and 3.

	DF 1	DF 2	DF 3	DF 4	DF 5	DF 6	DF 7
% variance explained	43.6	22.9	12.9	7.6	6.5	4.3	2.2
Canonical loadings							
Sq	−0.28	0.29	0.26	−0.14	−0.23	−0.02	−0.06
Ssk	−0.08	−0.17	0.15	0.00	0.00	0.24	−0.15
Sku	0.12	0.02	0.03	−0.02	0.26	−0.19	0.14
Sp	−0.26	0.14	0.54	0.01	−0.08	−0.07	−0.29
Sv	−0.10	0.42	0.23	−0.10	0.06	−0.04	0.05
Sz	−0.18	0.37	0.39	−0.07	0.01	−0.06	−0.08
Sa	−0.28	0.29	0.25	−0.15	−0.25	0.00	−0.07
Smr	0.16	−0.10	−0.52	0.05	0.09	0.22	0.47
Smc	−0.30	0.28	0.27	−0.15	−0.23	0.03	−0.02
Sxp	−0.22	0.28	0.17	−0.06	−0.25	−0.09	−0.06
Sal	−0.08	−0.02	−0.04	0.14	0.11	0.05	−0.32
Str	0.01	0.15	−0.21	0.00	0.13	0.04	−0.29
Std	−0.03	0.02	0.09	0.14	−0.16	−0.04	0.42
Sdq	−0.13	0.69	0.33	−0.06	−0.14	−0.04	0.11
Sdr	−0.21	0.67	0.32	−0.04	−0.13	−0.11	0.12
Vm	−0.17	0.00	0.33	−0.1	−0.01	0.06	−0.21
Vv	−0.30	0.27	0.28	−0.15	−0.22	0.03	−0.03
Vmp	−0.02	0.00	0.28	−0.10	−0.02	0.03	−0.28
Vmc	−0.29	0.29	0.21	−0.14	−0.27	0.01	−0.05
Vvc	−0.30	0.26	0.28	−0.16	−0.22	0.05	−0.03
Vvv	−0.22	0.28	0.17	−0.04	−0.21	−0.10	−0.03
Spd	0.19	0.08	−0.08	0.17	−0.02	−0.09	0.32
Spc	−0.17	0.45	0.37	0.34	−0.16	0.26	−0.05
S10z	−0.20	0.31	0.35	−0.22	−0.10	−0.09	0.01
S5p	−0.23	0.06	0.20	−0.26	0.12	−0.19	−0.09
S5v	−0.10	0.36	0.31	−0.11	−0.20	0.02	0.08
Sda	−0.12	−0.01	−0.16	−0.08	0.09	−0.04	−0.06
Sha	−0.10	−0.12	−0.26	−0.15	0.03	0.22	−0.41
Sdv	−0.12	0.09	0.04	0.03	0.05	−0.20	0.08
Shv	−0.14	0.06	−0.11	0.06	−0.13	−0.02	−0.15
Iso	0.01	0.15	−0.21	0.00	0.13	0.04	−0.29
1D	0.02	−0.15	0.00	0.21	−0.29	−0.03	0.15
2D	0.00	0.07	0.01	0.14	0.19	0.17	0.14
3D	0.22	0.19	−0.06	0.09	−0.14	0.03	0.03
WS	−0.07	−0.07	0.08	−0.20	−0.13	0.03	0.07
NS	0.48	−0.07	−0.01	−0.13	−0.12	0.30	0.01
LP	0.12	0.57	−0.08	0.29	0.04	−0.26	−0.14
SP	0.03	0.10	0.09	−0.04	0.00	−0.06	0.00

**Figure 9.**
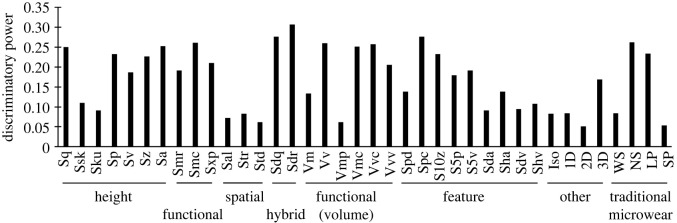
Total discriminatory power of each variable resulting from the total evidence analysis of raw data where ISO and TM data are combined.

Finally, the total discriminatory power of each variable is shown in figure 9. ISO parameters from height, functional, hybrid, functional (volume) and feature are among the most discriminating variables, whereas spatial and other families showed lower potential. TM variables, NS and LP, have high discriminatory powers. ISO variables that have high discriminatory value also tended to have strong correlations with frequencies of scratches and pits.

## 4. Discussion

### 4.1. ISO 25178-2 parameter performance

The Dqs treatment resulted in the vast majority of differences found among the ISO parameters between the control and remaining treatments and we, therefore, conclude this treatment produced the highest degree of change in surface texture. The large number of ISO parameters and their complexity makes it more difficult (compared with simple TM data) to describe the surface texture differences in simple terms. Here we endeavour to discuss the ISO parameters and describe the surface texture differences between the Dqs and P treatments. While other dietary treatments could have caused subtle microwear changes, the paucity of statistically significant results involving the other treatments indicates that our study was not capable of identifying these differences. As far we can ascertain, the other treatments were not differentiated from the control P. Therefore, there seems little value in discussing differences between the treatments other than between Dqs and P.

The areal field parameters defined by ISO 25178-2 are categorized into families that allow for easier conceptualization [73] (table 1). The Dqs treatment yielded higher values for numerous parameters from the height, functional, hybrid, volume and feature families. Height parameters are measures of the height of the surfaces and the distribution of the heights of those peaks. Sq (the root mean square height) and Sa (the arithmetic mean height) are similar measures and are closely correlated because they are essentially the same values only varying in how they are measured against the mean plane [74]. Sa and Sq are more stable despite orientation effects than parameters measuring maximum surface heights like Sz, Sp and Sv [75]. Nonetheless the Dqs surfaces yielded higher mean and absolute heights suggesting more amplitude of relief on these surfaces.

Functional parameters are derived from the height distribution measured from the mean plane, with Smr being a percentage of the surface area at a specified height (1 µm under the highest peak) and Smc being the inverse of that (*p* = 10%). Sxp (extreme peak height; *p* = 50%, *q* = 97.5%) is a measure of the height of the highest peaks from the mean plane (minus the most extreme peaks that may be extraneous). Sxp is a measure of the highest parts of the surface, measured from the mean plane, but excluding 2.5% of the highest peaks that might not be significant. Sp is a measure of the highest peak from the mean plane, which can include random extremely high points. Because Sxp is a measure of the highest peak with just this exclusion of 2.5% of the highest peaks, we would expect Sxp to have a similar relationship to dental wear surfaces as Sp. Dqs specimens are more extreme in the height of the peaks from the mean plane and this is again explained by a greater amount of absolute relief in the surface texture of this treatment.

Spatial parameters are measured from an autocorrelation plot, which is a measure of the periodicity of the surface texture from centre to periphery in a way that can allow us to measure how it changes from centre to edge and be used to describe its isotropy. Sal (the autocorrelation length) is the distance from the original location at which one finds a texture that is statistically different from it. Str (texture-aspect ratio) is a ratio of the minimum radius to the maximum radius (in the x/y plane) of the central lobe of the autocorrelation plot. Because the autocorrelation plot is a way of creating a spatially arranged ideal of the changes in texture throughout the surface, the asymmetry of this central lobe is a strong indicator of the isotropy of the surface texture. Std (texture direction) is the result of a Fourier transform of the surface and identifying the direction of the greatest power function in that transform to identify a privileged direction. This is essentially the orientation of the mean direction for the autocorrelation plot. The autocorrelation plot makes it more difficult to translate this easily into what we would expect in terms of common indentation patterns seen in TM, though as a set of measures of isotropy of texture (as well as orientation), this should be useful. Dqs was not significantly different in any spatial parameters, which suggests that the heterogeneity of the surfaces from one side to the other was not meaningfully different after this dietary treatment than it was in any other. Heterogeneity in the regions of each observed area would be more likely if mastication patterns dramatically changed with diet, perhaps in situations where a uniform set of parallel scratches from translational movements were replaced with random deep pits from orthal chewing. But in that case we would expect that change to coincide with the different food textures (all dough versus all pellet diets) and also be reflected in similar changes in orientation parameters.

The two-hybrid parameters (Sdq and Sdr) vary in what they measure and should be treated independently. Sdq, the root mean square gradient, is a hybrid parameter that accounts for the slope of points for the whole surface, essentially representing the angularity of the faces of the surface as a whole (a flat surface has Sdq = 0, a surface where the average slope is 45° has Sdq = 1) [73]. This would not take into account the height of elevated and indented surfaces, only the tilt, and would probably be a good measure of the angularity of the surface. The surfaces of the Dqs treatment have more tilted surfaces overall. This result is consistent with greater overall relief. Sdr, the developed interfacial area ratio, is essentially a comparison of the surface area that is added to what it would be if the surface was perfectly flat (think of a wrinkled piece of paper that is flattened out and increases the surface it covers), and is considered a measure of complexity [73]. Because the Dqs dietary treatment resulted in a surface that is so elevated and irregular, it also makes sense that it would result in a significantly increased Sdr because the surface area of those greater peaks and valleys is large.

Volume parameters are measures of the volumes of spaces above or below the surface, referred to as material (below the surface, as the material being worn) and void (above the surface, as the space left after material is worn away). There is a general measure of the material volume (Vm) or void volume (Vv) that are measured with respect to material ratio (mr), which is derived from Smc. There is also a set of parameters for materials and voids with respect to two calculated material ratio values, the bearing ratios (mr1 and mr2). The upper bearing ratio is set by default at 10%, and this divides the peak material volume (Vmp) from the core material volume (Vmc). The lower bearing ratio is set by default at 80%, dividing the core void volume (Vvc) from the dale void volume (Vvv). While Vm and Vv distinguish the volumes of surface above and below a horizontal plane determined by the material ratio, Vmp best represents the majority of the material that would be removed or plastically deformed when in contact with another surface in any translational movement, and Vmc could be the remaining material that would cause friction. The parts of the surface contributing to Vvc are likely to not be in contact with another surface, and the surface of regions measured by Vvv are likely to be uninvolved in anything besides containing materials broken off during wear. Dqs has distinctly different values for these parameters because the topography of these surfaces had such greater *z*-values, making the volumes larger. This is similar to the values seen with heights, but are likely to be larger values because volumes grow cubically.

Feature parameters are measures of motifs (dales and hills) that are formed by segmentation [76]. Sha, the mean hill area, is a feature parameter that describes the average area of the hills of each motif. If the majority of hills of a surface are of similar size, they are likely to have similar Sha values. It is unclear why other aspects of a motif are not significant, except perhaps that hills represent the swarfs and spaces between pits and scratches, which may be more similarly polished to a smooth surface in all teeth, leaving the indentations to vary more. The indentations, as scratches and pits, would have a greater variability. Dqs has a greater variety of motifs because of the greater relief it exhibits. Not only is there greater relief, but there are more motifs within motifs because of this increase in complexity.

Other (isotropy and directional) parameters are measures of the orientation of faces of the surface within the mean plane. One can readily imagine a set of parallel scratches that extend across the surface from the top to the bottom of the image, all being measured as facing 90° to the horizontal plane. These four parameters (isotropy, first, second and third directions) amount to an average orientation (isotropy) and the means of the three largest clusters of orientations. It is easy to imagine that a very scratched surface is more likely to have a greater number of parallel surfaces, and therefore lower isotropy, as long as the orientation maintained a uniform direction. Likewise, if orientation changes because of a change in masticatory behaviour (such as propalinal to translational movements) one might expect a significant change in direction parameters, but not isotropy. It may be that the generally greater amount of damage to the surface seen on the Dqs teeth accounts for the greater irregularity of the orientations and that the remaining dietary treatments are all more consistently covered in indentations that follow typical masticatory movements that rats normally exhibit.

### 4.2. Comparison of ISO and TM results

Because ISO-based DMTA is becoming more widely used for analysis of dental microwear (e.g. [–9,36,56,60,77]) it may be helpful to compare it with more easily conceptualized TM variables. Also, experimental tooth wear studies most often generate microwear structures that are caused by shear and compressive forces and continue to be characterized and described as scratches and pits. It is therefore important to understand how ISO data relate to scratch and pit features as described in experimental tooth wear studies [9]. Microwear is the product of the dynamic interplay of ingested particles, the occluding enamel and the force vectors recruited during mastication. TM deals only with discrete microwear features that are readily visible at the scale of analysis [70]. Scratches require both vertical and translational occlusal pressures exerted on an ingested particle that is capable of damaging enamel. Pits can be generated by the same particles, but require only vertical occlusal pressures. TM records the occurrences of individual microwear features. These features are dependent on each other only in the sense that they cannot occupy the same areas, often making them overlap, obliterate, or truncate each other, making individual indentations difficult or even impossible to recognize if they are too densely distributed. On the other hand, ISO parameters do not recognize individual indentations and are measures of identical surfaces based on the same three-dimensional data points. Some ISO parameters are measures of different subsets of those points (for example, peak volume and void volume), but the determination of the compositions of those subsets is dependent on the whole.

TM found significant differences between the pellet control (P) and three feeding treatments (Pde, Dcc, Dde) involving differences in pit frequency, and for one treatment (Dqs) related to scratch frequency. ISO, on the other hand, overwhelmingly suggested only one group (Dqs) as having been most dramatically altered. Dqs had the least amount of overlap with all other groups in multivariate space. ISO may be more sensitive to elongate microwear structures (scratches) and less prone to detect differences in pitting. ISO accounts for the entire scanned surface and scratches can include proportionally much more of the area scanned for each feature counted compared with pits. This suggests ISO may be more valuable for differentiating diets in groups such as ruminants that are largely differentiated by scratch frequency but may be less sensitive to differences relating to pitting (e.g. fruit browsing, hard object feeding) [8,78–83]. However, we caution that the microwear patterns produced by this experiment are a limited subset of all possible microwear textures. Studies involving microwear textures beyond what was produced in this study may produce different results.

Although TM methods are being replaced by technically superior DMTA methods, the frequencies of discrete microwear features continue to be good discriminators of microwear patterns and discriminant function analyses were most powerful when TM and ISO data were combined. It is difficult to pinpoint individual TM or ISO variables as being very strongly informative in these data. The best variable (Sdr) had only a modest discriminatory power (0.34) (figure 9) and numerous other ISO parameters and TM variables performed similarly. It is the combination of these parameters in multivariate discriminant function analysis that performed best in differentiating microwear patterns resulting from the feeding treatments. Nonetheless, ISO parameters that were strongly correlated with TM data had higher discriminatory powers than parameters with weaker correlations with TM data (figures 8 and 9). The ISO parameter Vmp (peak material volume) is the single notable exception to this pattern. It is significantly correlated with NS (narrow scratches), but was a weak discriminator of the resulting microwear textures. The nature of the correlations between TM and ISO variables are difficult to unravel.

### 4.3. How do the microwear patterns differ between treatments?

Prior to the 15-day feeding trials, all rats were fed the standardized pellet diet, without mineralogical additives. Therefore, it is best to discuss the relation of the microwear results and the feeding treatments in terms of deviation from the pattern shown by the control group (P) who continued to be fed the standard pellet diet.

The P group produced rather coarse microwear textures with high numbers of TM features (figure 5). The rats fed plain dough (D), which lacked hard or abrasive particles, experienced a minor reduction in the average number of TM microwear features, although no significant differences in ISO or TM data between the pellet and dough groups were found. There are two possible explanations for the insignificant differences: (1) dough generated no appreciable dental wear by itself over the 15-day feeding period, and the microwear features remaining on the teeth of the dough group were left over from the pellet diet fed to them before the 15-day trial, or (2) the microwear of both groups (P and D) were caused by attrition and unrelated to the specific nature of the diet.

The addition of the three mineralogical abrasives (calcium carbonate, diatomaceous earth and quartz silt) had varying effects on the microwear and strongly suggest the minerals themselves not only cause differential microwear but that they have different effects depending on the type of food. The addition of mineral abrasives to the dough diet resulted in greater changes than when added to pellets. Therefore studies, such as Merceron *et al*. [39] that report no effects when abrasive agents are added to food may be results that are contingent on the nature of the food itself.

Calcium carbonate powder, the softest of the three additives, had little effect on microwear. The Dcc treatment seems to have resulted in significant reduction of pits (LP) and change in one ISO parameter (Sha) and its shift away from the pellet control in multivariate space is seen in figure 7. Diatomaceous earth led to a more significant decrease in the number of pit features (LP and TP) when mixed with both pellets and dough, although again the effect appears to have been strongest when paired with the dough group (Dde). The most dramatic results are those related to quartz silt, the hardest and coarsest grained of the three abrasive additives. Introduction of quartz had no effect on the microwear of the pellet-fed group (Pqs). The Pqs group overlaps the P group extensively in multivariate space. However, the introduction of quartz silt to dough (Dqs) produced heavily modified microwear. Scratches were reduced in frequency by 50% and more than half of the ISO variables differed between P and Dqs. Dqs has the least overlap with P or any of the other treatments in multivariate space. These results are similar to those of Hoffman *et al*. [10], where sand added to the diets of sheep was associated with an increase in pits, but not scratches. In our results, changes involved either scratches or pits but never both types of variables.

These specific outcomes are not strictly consistent with what we might have anticipated. For instance, one might expect the introduction of very hard quartz silt particles to result in increased numbers of microwear features rather than fewer. Likewise, it may be difficult to explain why some treatments led to reductions in pits whereas others led to reductions in scratches. In palaeodietary studies, it is commonly assumed, for organisms that adopt a strong labio-lingual or mesio-distal translational vector in their masticatory stroke, heavily scratched wear surfaces are indicative of biogenic (phytoliths) or geological (dust, sand) abrasives [3,36,84]. The reduction of scratches associated with the addition of silt to any otherwise soft and non-abrasive dough diet, and the reductions in the numbers of pits associated with other diets, may be explained by differential behavioural responses of the rats to different food textures, including changes to occlusal pressures and force vectors. The shapes of indentations are the results of movements perpendicular, oblique, or parallel to the occlusal surface. Rats, like humans, avoid injury and unpleasant experiences, including the ingestion of hard and abrasive particles that can damage enamel. Most vertebrates avoid traumatic tooth fractures by modulating mastication patterns and pressures using oral sensory cues from the tongue, teeth and muscles of mastication [85–87]. It is possible that behavioural variation in patterns of mastication as a response to the sensory experience caused by different food textures is the underlying cause of some of the differences in dental microwear [88]. But rats and other murids are consistent in their propalinal masticatory patterns [89–91], making the most likely effect of food texture being one in which mastication per bolus of food is primarily the result of changes to food texture rather than behavioural modifications to mastication vectors other than regulation of bite force when abrasives are sensed.

## 5. Conclusion

Many ISO parameters are correlated to pit and scratch frequencies, the main variables of traditional dental microwear analysis, suggesting that ISO-based DMTA is useful for characterizing dental wear textures in ways that may capture the same phenomena that TM methods do. We attempted to control for the abrasive effects of food by adding abrasive agents to a soft non-abrasive dough, in addition to the harder more brittle pellet diet that is normally fed to rats. The pellet diet, which generates a coarse type of microwear texture on its own, largely obfuscated the effects of the mineralogical abrasives. By contrast, the abrasives had clear differential effects when the abrasive nature of the food itself was minimized by the dough diet. Softer and finer-grained abrasive agents (calcium carbonate and diatomaceous earth) caused changes relating to pit frequency. ISO parameters were largely insensitive to these changes. On the other hand, quartz silt had an impact on scratch frequency and this change was abundantly recognized by ISO parameters. *In vivo* microwear studies that expose animals to a variety of abrasive agents show no association of particular dental wear patterns with particular dietary abrasives. This may be due to a variety of animal models, experimental conditions, food types and abrasive agents. However, our results here suggest that the effects of abrasive agents are co-dependent on properties of the food itself. *In vivo* dental microwear is not only dependent on the material properties of ingested particles and teeth, but it is dependent on variation that animals may have in their behavioural response to apparent food texture. The effects of these variables may be difficult to unravel or predict.

Attempts to control the role of abrasives and food texture in dental microwear formation has its limitations, which we hope can be seen as opportunities for identifying new questions. In combination with cineradiography and XROMM, questions about the role of mastication rates, patterns of movement, chewing duration and how these are modified with different food textures could be investigated. Further information about the material properties of food items at a variety of scales could help explain how that relates to any changes in behaviours that result in wear. And, as the goal is to not only atomize the understanding of how this system works but also to apply this to the study of palaeoecology of extinct organisms, the natural next steps are to apply similar asessments to the natural experiments of evolution among modern wild taxa.

## Data Availability

The data are provided in electronic supplementary material [93].
